# Correlation analysis between physical activity and depressive tendencies among occupational groups: an isotemporal substitution approach

**DOI:** 10.1186/s12889-023-17134-0

**Published:** 2023-11-14

**Authors:** Yihua Liu, Haoxiang Lin, Hao Zhang, Xiaoyue Zhang, Shengli Yin

**Affiliations:** 1 Department of Social Medicine and Health Education, School of Public Health, Peking University, Beijing, China; 2 Institute for Global Health and Development, Peking University, Beijing, China; 3 DeZhou Center for Disease Control and Prevention, Dezhou, China

**Keywords:** Physical activity, Depressive tendencies, Isotemporal substitution model, Occupational groups

## Abstract

**Objectives:**

Sedentary behaviour (SB) and physical activity (PA) have been shown to be associated with depression. However, behaviours, such as PA, occupy a portion of an individual’s 24-h day. Thus, an increase in time for one behaviour takes away time from another. Previous evidence suggests that it would be more appropriate to shift the focus to the importance of reallocating time spent in sedentary behaviour to time spent in physical activity. The aim of this study was to analyse the mutual replacement effect of different health behaviours on depressive tendencies by isotemporal substitution modelling (ISM) under the objective condition of considering a limited 24-h day. Second, we aimed to further explore the potential association between excessive or insufficient sleep duration and depressive symptoms.

**Methods:**

A total of 10656 employees from 79 companies in four provinces of China participated in this survey.

The Center for Epidemiological Studies Depression Scale (CES-D) was used to measure workers’ depressive tendencies. The duration of various types of physical activity was self-reported by workers based on the International Physical Activity Questionnaire (IPAQ). ISM was used to assess the associations of time spent in different activities on displacement of equivalent time spent on other activities with depression risk.

**Results:**

A total of 10656 participants (89.5% of the sample) were included in the analysis.

The ISM found that a 30-min unit of SB replaced with walking (OR, 95% CI: 0.83, 0.77–0.88), sleep (≤ 8 h) (OR, 95% CI: 0.77, 0.74–0.79), moderate physical activity (MPA) (OR, 95% CI: 0.87, 0.81–0.93) and vigorous physical activity (VPA) (OR, 95% CI: 0.91, 0.84–0.99) was significantly and negatively associated with the risk of depressive tendencies. When sleep duration was less than 8 h, each additional half hour of sleep time was significantly associated with a lower risk of depressive tendencies, and this association was no longer significant after 8 h.

**Conclusion:**

Prolonged SB is common in the current workplace in China. Replacing an average of 30 min per day of SB with VPA and MPA, even walking is associated with less depression among workers. In addition, insufficient daily sleep is also an important risk factor for workers' depressive tendencies. These findings provide valuable evidence to promote mental health among occupational groups and support the development of healthy workplaces.

## Introduction

Approximately 300 million people worldwide are affected by depression [1]. The Global Burden of Disease study showed an almost 50% increase in the annual incidence of depression from 1990 to 2017 [2]. Depression is associated with many serious health-related disorders, such as cardiovascular disease and premature mortality [3, 4], and it poses an enormous potential economic burden to society [5].

Depression is a complex mental health disorder that results in patients with the same diagnosis of depression having different symptoms, which also poses an enormous challenge for the treatment of depression [6]. Previous studies have found that the actual treatment effects of either pharmacotherapy [7] or psychotherapy [8] tend to be small to modest. Pharmacotherapy can also cause several adverse side effects, such as headaches and nausea [9]. Therefore, the prevention of depression based on healthy behaviours, such as promoting physical activity (PA) or decreasing sedentary behaviour (SB), has gradually become a public health priority [10].

### Evidence prior to this study

Increasing evidence suggests that regular PA, especially moderate-to-vigorous physical activity (MVPA), is associated with less depression among adults [11]. PA not only has a significant direct antidepressant effect based on biological evidence from humans or animals (e.g., associated with structural abnormalities and dysregulation of some neuroplastic mechanisms) [12] but also indirectly reduces the risk of mental disorders by enhancing physical fitness and function (e.g., enhancing the body's cardiorespiratory fitness) [13] and is a useful addition to the clinical treatment of mental health problems.

Recently, increasing concern about prolonged SB leading to various health problems has grown [14]. A sizeable review has established that SB leads to an increased risk of depression in adults [15, 16]. The conclusion of a recent prospective study demonstrated that mentally passive SB could increase the risk of depression in the future (RR = 1.10, 95% CI 1.03–1.19) [17]. Considering the widespread occurrence of sedentary habits within occupational groups such as office workers, it becomes imperative to investigate the association between SB, PA, and depression within this population [18, 19]. The data show that for certain occupational groups, working hours were mostly sedentary (77.0%, 95% CI: 76.3, 77.6), and there were significant differences between their working hours and nonworking hours [20]. Gaining insights into the underlying factors driving the association can facilitate the creation of focused interventions and proactive strategies aimed at reducing the risk of depression in this specific population.

In addition, the quality and duration of sleep are potential determinants of depression [21]. Based on the data from a review of prospective studies, compared with normal sleep duration, too much sleep (RR = 1.42, 95% CI = 1.04 to 1.92) or too little sleep (RR = 1.31, 95% CI = 1.04 to 1.64) can lead to an increased risk of depression [22].

### Why research is required

First, behaviours, such as sedentary lifestyle, amount of exercise or duration of sleep, occupy a portion of an individual’s 24-h day. Thus, an increase in time for one behaviour takes away time from another. In other words, the previously reported associations between depression and its behaviour-related determinants (e.g., SB, PA, sleep) are mainly from a sort of statistical association, not a real replacement effect between different behaviours. This limitation can be overcome by the isotemporal substitution modelling (ISM) approach [23]. ISM simultaneously models the specific activity being performed and the specific activity being displaced in an equal time-exchange manner [23]. Thus, the model not only controls for the effects of other time-independent behaviours on the outcome variable but also captures the effects of temporal substitution between different behaviours. The models have revealed novel findings in various aspects, such as all-cause mortality or cardiovascular diseases [24, 25]. Evidence suggests that it would be more appropriate to shift the focus to the importance of reallocating time spent in sedentary behaviour to time spent in physical activity [26]. The application range of ISM is progressively widening. We aspire to utilize this model to conduct a more profound exploration of the correlation between different types of physical activities and depression, thereby establishing a robust basis for the refinement of advanced physical activity guidelines.

Second, the association between sleep duration and depression is controversial. Both excessive and insufficient sleep duration show a significant association with depression [22]. First, a considerable number of studies have demonstrated the bidirectional relationship between sleep disturbances such as insomnia, which results in a significant reduction in sleep duration and an increase in depressive symptoms [27]. However, hypersomnia is prevalent and persistent across mood disorders, which leads to a significant increase in sleep duration among people with depressive symptoms [28]. Therefore, both excessive and insufficient sleep durations that lead to an increased risk of depression require further investigation.

Third, although the antidepressant effects of exercise have been widely demonstrated, prior research has indicated a contentious association between different exercise intensities and depression. A review found a stronger correlation between high-intensity exercise and depression in observational studies, while intervention studies did not find significant differences in depression outcomes across distinct exercise types [29]. Furthermore, prior research has commonly focused on assessing the direct association or effect of physical activities of different intensities on depression. There has been limited attention to investigating their potential mutual substitution effects. This question warrants further exploration with the support of ISM.

### Objectives

In summary, this study expects to resolve the following issues based on a large sample of occupational groups from multiple cities in China. First, under the objective condition of considering a limited 24-h day, a thorough analysis of the mutual replacement effect of different health behaviours on depressive tendencies was conducted using ISM. Second, we explored the potential association between excessive or insufficient sleep duration and depressive symptoms.

## Methods

### Settings

The Asia Best Workplace Mainland China (ABWMC) program was a cross-sectional survey to support companies in building healthy workplaces through policy, infrastructure, culture and healthy employees. The ABWMC program was designed by Peking University and organized by the American International Assurance Company. We invited companies to join the program using a purposive selection method. The inclusion criteria for participating companies were as follows: (1) legal companies registered in China; (2) at least 100 full-time employees; and (3) agreement to participate in the program [30].

### Sampling

The analyses were based on data from the 2021 ABWMC program. We recruited a total of 79 companies in four provinces: Shanghai, Jiangsu, Guangdong and Beijing. The total sample size was 11,903. The human resource departments of each company delivered the questionnaires to all employees. All employees who were (1) aged 18 years old or above and (2) full-time employees were invited to participate in this program.

### Data collection

Experts at Peking University designed standardized questionnaires, including sociodemographic information, PA-related behaviour and other covariates. Then, we generated an online questionnaire system and specific questionnaire links based on the Ipsop. All questionnaire completion logic (e.g., skip questions or mutually exclusive options) was set through the online questionnaire system, and a function was set to exclude certain unqualified questionnaires (e.g., questionnaires with less than 3 min of answer time were automatically excluded) to guarantee the quality of the data. The human resources departments of each participating company delivered the internet link to all employees. Participants were required to read the online informed consent form before starting to answer. All data collected were examined by the researchers at Peking University, and the respondents were contacted for clarification if any problems were detected. The study was approved by the Peking University Health Sciences Center Ethics Committee (IRB00001052-21086). All methods were performed in accordance with the relevant guidelines and regulations.

### Measurement

#### Depressive tendencies

The Center for Epidemiological Studies Depression Scale (CES-D) was used to measure workers’ depressive tendencies [31]. We used the revised Chinese version (CESD-9) with 9 questions [32]. Participants will be asked to recall how often the feelings described in each item occurred in the past week. The scoring is as follows: less than 1 day = 0, 1–2 days = 1, 3–4 days = 2, and 5–7 days = 3. All items included four response categories indicating the frequency of depressive symptoms. Of the nine items, seven focused on positive symptoms, while the other two (items 5 and 8) assessed negative symptoms of depression. A score is assigned by combining all items (after reversing the positive mood items). A total of 27 points on the scale with a score of 10 or more indicated depressive tendencies. The scale has been tested in previous studies in large Chinese populations, with a Cronbach’s α coefficient of 0.95 and high scale sensitivity [32]

#### Physical activity

PA behaviour was measured using the Taiwanese short version of the International PA Questionnaire (IPAQ) [33]. Respondents were asked to recall the frequency and duration of walking and moderate and vigorous PA in the past 7 days. We calculated the total time employees spend each week exercising at each level of intensity (frequency*duration) and then calculated the average daily PA time (divided by 7). Additionally, we evaluated the overall physical activity level of each individual based on their weekly duration of PA and corresponding MET values for each type of PA (walking: 3.3 METs/min, MPA: 4.0 METs/min, VPA: 8.0 METs/min). According to the sum of the MET values of each type of PA, the physical activity level of an individual was classified as low (< 600 MET-min/wk), moderate (600–1500 MET-min/wk) or high (> 1500 MET-min/wk) [33].

#### Sedentary behaviour

The duration of SB was also self-reported by workers based on the IPAQ questionnaire on average daily SB time [33]. We asked respondents to recall the average daily time spent in 1) sitting time while working or studying, including writing, operating a computer at work, and answering the phone; 2) recreational sitting time after work, including resting, reading, playing on the phone or computer and chatting; and 3) time spent driving or taking transportation in the past week. The sum of the three types of static sitting time was considered the average individual daily duration of SB. In the descriptive analysis, we categorized sedentary behaviour based on the accumulated sitting time per day into two groups: normal (≤ 8 h/day) and prolonged (> 8 h/day).

#### Sleep duration

Participants were asked to recall the average number of hours of sleep per day over the past week. We defined eight hours of sleep as sufficient sleep, while the opposite was considered insufficient sleep.

#### Potential confounders

The potential confounders included sex, age, educational level, marital status, occupation type and job position, body mass index (BMI) and smoking status (current smoker or nonsmoker). BMI was categorized into three groups: normal (< 24), overweight (24–28), and obese (> 28). We put all these variables into the regression model to control for their confounding effects on depression tendencies.

### Data handling

The ISM assumes that an increase in the duration of one behaviour in one day leads to a decrease in the time spent on another behaviour. Therefore, we adopted the following data cleaning procedures to ensure the reliability of the analysis results. According to the guidelines for data processing and analysis of the IPAQ, [34] the outliers of time spent per day for different types of behaviours (e.g., SB time, calculated as three times the standard deviation, with a lower limit of 3.8 and an upper limit of 18) were filtered by the data exploration function, and then the outliers of walking, MPA and VPA time (< 10 min/day) were recorded as 0, the extreme outliers of sleep were recorded as 7, and SB were recorded as missing. Afterwards, the sum of each independent time was calculated. Considering the recall bias associated with self-reported questionnaires and concerns about data stability, we excluded individuals with total activity time less than 12 h and greater than 28 h, resulting in a final sample size of 10656 (89.5%) individuals. The data cleaning was conducted as follows:

- SB time for outliers (*N* = 36)
- Total time > 28 h (*N* = 149)
- Total time < 12 h (*N* = 1062)

Final Analytic Dataset, *N* = 10656 (89.5%).

### Statistical analyses

The depressive tendencies of the participants were first described by number and frequency. The chi-square test was used to detect significant differences among different groups.

Subsequently, in the multivariate analysis, we initially conducted traditional logistic regression analysis as model 1, which incorporated duration of sleep, SB, MPA,VPA, and other covariates such as job position and age, to calculate the independent effect of each behaviour on depressive tendencies.

Model 1 is shown as follows:

$$\mathrm{Logit}\left(\mathrm{P}\right)= {\upbeta }_{0}+ {\upbeta }_{1}{\mathrm{X}}_{1} \left(\mathrm{sitting}\right)+ {\upbeta }_{2}{\mathrm{X}}_{2} \left(\mathrm{Sleep}\right)+ {\upbeta }_{3}{\mathrm{X}}_{3} \left(\mathrm{walking}\right) + {\upbeta }_{4}{\mathrm{X}}_{4} \left(\mathrm{MPA}\right)+ {\upbeta }_{5}{\mathrm{X}}_{5} \left(\mathrm{VPA}\right)+ {\upbeta }_{6}{\mathrm{X}}_{6} \left(\mathrm{other covariates}\right)$$

The coefficient β for one type of activity represents the effect of increasing this type of activity while holding the other activities constant in this model.

ISM was then used to assess the associations of time spent in different activities in displacement of equivalent time spent on other activities with depression risk. This model assumes that the time consumed by an individual for any behaviour during a fixed 24-h day results in an isochronous switch to another behaviour, while the total time for both behaviours remains constant [35]. For example, to estimate the effect of replacing SB with 30 min of walking, SB needs to be removed from the model based on the total time of the behaviour remaining constant.

Model 2 is shown as follows:

$$\mathrm{Logit}\left(\mathrm{P}\right)= {\upbeta }_{0}+ {\upbeta }_{2}{\mathrm{X}}_{2} \left(\mathrm{sleep}\right)+ {\upbeta }_{3}{\mathrm{X}}_{3} \left(\mathrm{walking}\right)+ {\upbeta }_{4}{\mathrm{X}}_{4} \left(\mathrm{MPA}\right) + {\upbeta }_{5}{\mathrm{X}}_{5} \left(\mathrm{VPA}\right)+ {\upbeta }_{6}{\mathrm{X}}_{6} \left(\mathrm{other covariates}\right)+ {\upbeta }_{7}{\mathrm{X}}_{7} \left(\mathrm{Total}\right)$$

The coefficients β in this model represent the effect of a 30-min substitution of SB with one of the activity types (LPA or MVPA) while holding the other activity types and total wear time constant.

All statistical analyses were conducted using SPSS software version 22.0, and the level of significance was set at *p* < 0.05.

## Results

A total of 10656 participants (89.5% of the baseline sample) were included in the analysis. There were no significant differences between excluded and included workers in age, sex, occupational status or prevalence of depressive tendencies. Table 1 shows the characteristics of the study participants. Participants were aged 18 to 57 years and had a mean age of 33.1 years. More than half of the participants were office workers. The average daily SB time of workers is 10.4 h, and the average daily time spent on PA of different intensities is less than half an hour.

**Table 1:** Sociodemographic characteristics of study participants (*N* = 10656)

Items		n (%) or M ± SD
Gender	Male	4733 (44.4)
	Female	5923 (55.6)
Age/years		33.06 ± 6.92
Education level	College degree and below	2698 (25.3)
	Undergraduate and above	7958 (74.7)
Occupation	Office workers	7464 (70.0)
	Others	3192 (30.0)
Positions	General Staff	6563 (61.6)
	Management	4093 (38.4)
SB time (hour/day)		10.44 ± 2.94
Sleep time (hour/day)		7.13 ± 0.93
Walking time (hour/day)		0.24 ± 0.36
MPA (hour/day)		0.14 ± 0.24
VPA (hour/day)		0.09 ± 0.21

*MPA* Moderate-intensity PA, *VPA* Vigorous-intensity PA

Occupation*: Office workers refers to individuals working in office environments, such as white-collar workers or administrative personnel

Positions *: General staff refers to nonmanagerial personnel within a company

Table 2 shows the number and prevalence of depressive tendencies stratified by the characteristics of the respondents. Of the 10656 workers who participated in the study, 1791 (16.8%) reported the presence of depressive tendencies. The male population showed a higher prevalence of depressive tendencies. The prevalence of depressive tendencies among office workers (17.8%) was significantly higher than that among others (14.8%). Depressive tendencies were more common in the obese (20.6%) and smoking (21.6%) groups.

**Table 2:** Depressive tendencies status of participants

Items		Depressive tendencies	
		n (%)	*P*
Gender	Male	853 (18.0)	Reference
	Female	938 (15.8)	< 0.01
Age/years	≥ 40	1722 (17.3)	Reference
	< 40	69 (9.6)	< 0.001
Education level	College degree and below	471 (17.5)	Reference
	Undergraduate and above	1320 (16.6)	0.296
Occupation*	Office workers	1329 (17.8)	Reference
	Others	462 (14.5)	< 0.001
Positions*	General Staff	1108 (16.9)	Reference
	Management	683 (16.7)	0.793
BMI	Normal	1260 (16.4)	Reference
	Overweight	408 (17.3)	0.434
	Obesity	123 (20.6)	< 0.001
Average daily sitting time	≥ 8 h	233 (13.3)	Reference
	< 8 h	1558 (17.5)	< 0.001
Average daily sleep time	≤ 8 h	762 (24.7)	Reference
	> 8 h	1029 (13.6)	< 0.001
Intensity of PA	Low	1222 (20.2)	Reference
	Moderate	338 (12.7)	< 0.001
	High	231 (11.8)	< 0.001
Smoking	No	1419 (15.9)	Reference
	Currently smoking	372 (21.4)	< 0.001
Total		1791 (16.8)	

Table 3 shows the results for the independent effect and IS models after adjusting for covariates. In terms of independent effects, shorter daily SB time and longer sleep (≤ 8 h), walking and MPA time were protective factors against depressive tendencies. Increasing the time of VPA alone did not significantly reduce the risk of depressive tendencies; the ISM found that a 30-min unit of SB replaced with walking (OR, 95% CI: 0.83, 0.77–0.88), sleep (≤ 8 h) (OR, 95% CI: 0.77, 0.74–0.79), MPA (OR, 95% CI: 0.87, 0.81–0.93) and VPA (OR, 95% CI: 0.91, 0.84–0.99) was significantly and negatively associated with the risk of depressive tendencies. When sleep duration was less than 8 h, each additional half hour of sleep time was significantly associated with a lower risk of depressive tendencies, and this association was no longer significant after 8 h. Substitution between walking and PA of different intensities did not significantly change the effect on depressive tendencies.

**Table 3:** Independent and replacement effects of sitting, sleeping, walking, MPA and VPA on depressive tendencies risk (OR, 95%CI)

	With 30 min of:					
1. Replace 30 min of:	Sitting	Sleeping (≤ 8 h)	Sleeping (> 8 h)	Walking	MPA	VPA
Sitting		**0.77 (0.74–0.79)**^b^	1.01 (0.91–1.10)	**0.83 (0.77–0.88)**^b^	**0.87 (0.81–0.93)**^b^	**0.91 (0.84–0.99)**^a^
Sleeping (≤ 8 h)	**1.30 (1.26–1.34)**^b^			**1.08 (1.00–1.16)**^b^	**1.14 (1.06–1.22)**^b^	**1.23 (1.15–1.34)**^b^
Sleeping (> 8 h)	0.99 (0.90–1.10)			**0.82 (0.73–0.93)**^b^	**0.87 (0.77–0.98)**^b^	0.95 (0.84–1.07)
Walking	**1.21 (1.13–1.30)**^b^	0.93 (0.86–1.00)	**1.21 (1.08–1.37)**^b^		1.07 (0.98–1.17)	1.12 (1.00–1.24)
MPA	**1.14 (1.07–1.22)**^b^	**0.88 (0.82–0.95)**^b^	**1.15 (1.03–1.30)**^a^	0.96 (0.88–1.04)		1.08 (0.95–1.22)
VPA	**1.06 (1.01–1.19)**^a^	**0.59 (0.51–0.68)**^b^	1.06 (0.94–1.19)	0.92 (0.84–1.01)	0.92 (0.81–1.03)	
2.Independent effect	**1.04 (1.03–1.05)**^b^	**0.80 (0.75–0.87)**^b^	1.04 (0.95–1.15)	**0.86 (0.80–0.92)**^b^	**0.91 (0.85–0.97)**^b^	0.99 (0.92–1.07)

Adjusted for sex, age, educational level, marital status, occupation types, smoking status and job position

^a^represents *P* < 0.05

^b^represents *P* < 0.01

## Discussion

This study explored the association between SB, PA, sleep, and depression and how replacing these behaviours may influence depressive tendencies among occupational groups based on data from a cross-sectional survey of a larger sample from four provinces in China. Our results prove that an increasing duration of daily SB and a lack of exercise are associated with a higher risk of depression, as observed in our independent effect analysis. Although this explanation is valid, it is not practical because it does not consider the time displaced by SB due to the finite time of one day. Therefore, we conducted a further analysis of the replacement effect between various health behaviours on depressive tendencies.

### Displacement effects of SB on other behaviours

We observed a significant association between the duration of SB and the risk of depressive tendencies both for the independent effects and the replacement effect of SB on other behaviours. Our findings are consistent with some recent studies that demonstrated the favourable impact of reducing SB on depression. Scholars have proposed that the main mechanism of SB that leads to depression is the social withdrawal hypothesis at the mental health level, which suggests that prolonged use of electronic devices and other behaviours drive people away from other direct social interactions and physical activities, leading to an increased risk of detecting mental disorders such as depression [36]. According to a previous review, the average daily sedentary duration for employees is estimated to be approximately 9.4 h per day, a figure notably lower than our own findings of 10.4 h per day [37]. However, the assessment of sedentary behaviour in most surveys relies on self-report methods, introducing a certain level of inaccuracy due to factors such as recall bias, which makes direct numerical comparison challenging [38]. Our study primarily focused on the office working population, which spends a substantial amount of time sitting and working at desks each day [39]. These results still emphasize the need for increased attention and solutions aimed at addressing prolonged sitting behaviour among this specific group.

The association of PA and SB with health is more complicated. The intersection of movement-related behaviours and their implications for health outcomes has garnered significant attention [26]. The debate on whether increased exercise can attenuate the negative health effects of SB is still ongoing, and the conclusions are controversial [40]. A comprehensive review published in the Lancet revealed that adopting higher levels of daily physical activity can eliminate the heightened risk of all-cause mortality and cardiovascular diseases associated with prolonged sitting [41]. In our findings, replacing 30 min of SB each day with physical activity, regardless of intensity, was significantly negatively correlated with the risk of developing depressive tendencies. This result aligns with previous research conclusions, further emphasizing that exercise can mitigate the harms of prolonged sitting. In future interventions, combining both behaviours could potentially generate better health benefits.

### Different intensities of PA with depression

We found that substituting SB with various intensities of PA, including VPA, can significantly reduce the risk of depressive tendencies. In contrast, according to the odds ratio, walking and MPA seem to have more positive effects on depression. A randomized controlled trial found that the light PA level group reduced their depression score more than the moderate and vigorous PA level groups [42]. One possible explanation is that people with depression tend to prefer lighter exercises such as yoga or walking, which are helpful in adjusting their breathing and lead to greater self-esteem and feelings of mastery [43]. Comparing the results of the two models, the ISM identified significant findings that were not detected by the original linear regression model. The new model's ability to uncover previously unnoticed significant results highlights its enhanced sensitivity and capability to capture intricate relationships within the data. Traditional linear regression models might overlook subtle interactions that can be unveiled by the ISM [35]. Consequently, researchers and analysts should consider adopting such advanced models to extract deeper insights and make more informed decisions based on their data.

Recent studies have explored how replacing one activity with another affects health outcomes such as mortality and cardiovascular disease based on the ISM [44]. Contrary to previous research, the replacement effect between PA of different intensities on depressive symptoms in our study was no longer significant after controlling for covariates. A randomized controlled trial compared the effects of different PA intensities on posttreatment depression severity and found no significant differences among the LPA, MPA and VPA groups posttreatment [42]. The nonsignificant results might be indicative of a threshold effect, suggesting that a certain minimal level of exercise intensity is necessary to trigger notable changes in depressive symptoms [45]. However, the alternating substitution of high- and low-intensity exercises for relatively short durations each day may not have a significant impact on individual depression. Additionally, individual variability plays a substantial role in how people respond to different types of exercise intensities [46]. Future research could delve deeper into individual variations, long-term effects, and potential interactions between exercise intensity and other variables to provide a more comprehensive understanding of how different exercise intensities truly impact depression.

### Nonlinear association between sleep duration and depression

First, there was a significant positive correlation between shorter sleep duration and the risk of depressive tendencies when individuals slept less than 8 h/day, both as a direct effect and replacement effect to other physical activities. The mechanisms underlying the association between sleep duration and depression are still not fully understood. A clinical cohort study found that both short and long sleep durations predicted a poorer course in depressive disorders [47]. One underlying explanation for our findings is that only a short sleep duration (< 8 h/day) may increase the risk of depression. First, short sleep duration may increase daytime tiredness, which has been predictive of depression [48]. It is also possible that participants who reported depressive symptoms also suffered from sleep disorders. A study found that subjects with insomnia were more likely to remain depressed [49]. Another explanation is that shorter sleepers might simply have more time for pessimistic thoughts, thus contributing to their depression [48].

Interestingly, the effect of daily sleep duration on depression, whether direct or as a substitution, is no longer significant when it exceeds 8 h/day. This could imply that there exists an optimal threshold for sleep duration beyond which additional sleep does not significantly impact depression. First, excessive sleep duration has been significantly correlated with lower levels of physical activity, [50] which is a critical predictor of depressive symptoms [17]. Individuals who spend many hours sleeping may be unsatisfied with being unable to activate themselves.

### Implications

Our findings have implications. First, the results of the ISM revealed significant associations between VPA and depression that were not found in the original linear regression. This finding can provide innovative analytical thoughts for the identification of influencing factors that are more relevant to the real situation. Second, the survey report will be disseminated to the participating companies to provide them with recommendations on how to enhance the PA level of their employees. Moreover, the School of Public Health, Peking University Health Science Center, has many opportunities to attend training workshops, providing a good opportunity to showcase these findings to policy makers.

### Limitations

This study has several limitations. First, we used only cross-sectional data for estimation. The causal relationship between SB, PA, and depression cannot be inferred. Second, we used self-reported data for walking, MPA, VPA or SB rather than the more accurate tracker-based measurements, so participants may have overestimated PA levels. Third, the results could be biased because they are not based on a randomized study. However, as the participants were recruited from different parts of China and belonged to different types of companies, it is believed that the overall picture is meaningful.

## Conclusion

Prolonged SB is common in the current workplace in China. Replacing an average of 30 min per day of SB with VPA and MPA, even walking is associated with less depression among workers. In addition, insufficient daily sleep is also an important risk factor for workers' depressive tendencies. These findings provide valuable evidence to promote mental health among occupational groups and support the development of healthy workplaces.

## Data Availability

The data of the studies is accessible via Peking University, School of Public Health. The datasets used and/or analysed during the current study available from the corresponding author on reasonable request.

## References

[1] WHO (2017). Depression and other common mental disorders: global health estimates.

[2] Liu Q, He H, Yang J (2020). Changes in the global burden of depression from 1990 to 2017: findings from the global burden of disease study [J]. J Psychiatr Res.

[3] Correll CU, Solmi M, Veronese N (2017). Prevalence, incidence and mortality from cardiovascular disease in patients with pooled and specific severe mental illness: a large-scale meta-analysis of 3,211,768 patients and 113,383,368 controls [J]. World Psychiatr.

[4] Capuron L, Lasselin J, Castanon N (2017). Role of adiposity-driven inflammation in depressive morbidity [J]. Neuropsychopharmacology.

[5] König H, König HH, Konnopka A (2019). The excess costs of depression: a systematic review and meta-analysis [J]. Epidemiol Psychiatr Sci.

[6] Fried EI, Nesse RM (2015). Depression is not a consistent syndrome: An investigation of unique symptom patterns in the STAR*D study [J]. J Affect Disord.

[7] Cipriani A, Furukawa TA, Salanti G (2018). Comparative efficacy and acceptability of 21 antidepressant drugs for the acute treatment of adults with major depressive disorder: a systematic review and network meta-analysis [J]. Focus (American Psychiatric Publishing).

[8] Cuijpers P, Karyotaki E, Reijnders M (2019). Was Eysenck right after all? A reassessment of the effects of psychotherapy for adult depression [J]. Epidemiol Psychiatr Sci.

[9] Anderson HD, Pace WD, Libby AM (2012). Rates of 5 common antidepressant side effects among new adult and adolescent cases of depression: a retrospective US claims study [J]. Clin Ther.

[10] Schuch FB, Vancampfort D, Rosenbaum S (2016). Exercise improves physical and psychological quality of life in people with depression: a meta-analysis including the evaluation of control group response [J]. Psychiatr Res.

[11] Netz Y, Wu MJ, Becker BJ (2005). Physical activity and psychological well-being in advanced age: a meta-analysis of intervention studies [J]. Psychol Aging.

[12] Schuch FB, Vancampfort D, Firth J (2018). Physical activity and incident depression: a meta-analysis of prospective cohort studies [J]. Am J Psychiatr.

[13] Kandola A, Ashdown-Franks G, Stubbs B (2019). The association between cardiorespiratory fitness and the incidence of common mental health disorders: a systematic review and meta-analysis [J]. J Affect Disord.

[14] Biswas A, Oh PI, Faulkner GE (2015). Sedentary time and its association with risk for disease incidence, mortality, and hospitalization in adults: a systematic review and meta-analysis [J]. Ann Intern Med.

[15] Zhai L, Zhang Y, Zhang D (2015). Sedentary behaviour and the risk of depression: a meta-analysis [J]. Br J Sports Med.

[16] Teychenne M, Ball K, Salmon J (2010). Sedentary behavior and depression among adults: a review [J]. Int J Behav Med.

[17] Huang Y, Li L, Gan Y (2020). Sedentary behaviors and risk of depression: a meta-analysis of prospective studies [J]. Transl Psychiatry.

[18] Clemes SA, O’Connell SE, Edwardson CL (2014). Office workers' objectively measured sedentary behavior and physical activity during and outside working hours [J]. J Occup Environ Med.

[19] Reducing sitting time in office workers: Short-term efficacy of a multicomponent intervention [J]. Prev Med. 2013. 10.1016/j.ypmed.2013.04.004 23597658

[20] Thorp AA, Healy GN, Winkler E (2012). Prolonged sedentary time and physical activity in workplace and non-work contexts: a cross-sectional study of office, customer service and call centre employees [J]. Int J Behav Nutr Phys Act.

[21] Wichniak A, Wierzbicka A, Walęcka M (2017). Effects of antidepressants on sleep [J]. Curr Psychiatry Rep.

[22] Zhai L, Zhang H, Zhang D (2015). Sleep duration and depression among adults: a meta-analysis of prospective studies [J]. Depress Anxiety.

[23] Mekary RA, Lucas M, Pan A (2013). Isotemporal substitution analysis for physical activity, television watching, and risk of depression [J]. Am J Epidemiol.

[24] Stamatakis E, Rogers K, Ding D (2015). All-cause mortality effects of replacing sedentary time with physical activity and sleeping using an isotemporal substitution model: a prospective study of 201,129 mid-aged and older adults [J]. Int J Behav Nutr Phys Act.

[25] Wang YT, Liu HM, Cao SX (2022). Application of isotemporal substitution model in epidemiological research] [J]. Zhonghua liu xing bing xue za zhi = Zhonghua liuxingbingxue zazhi.

[26] Grgic J, Dumuid D, Bengoechea EG (2018). Health outcomes associated with reallocations of time between sleep, sedentary behaviour, and physical activity: a systematic scoping review of isotemporal substitution studies [J]. Int J Behav Nutr Phys Act.

[27] Krystal AD (2012). Psychiatric disorders and sleep [J]. Neurol Clin.

[28] Kaplan KA, Harvey AG (2009). Hypersomnia across mood disorders: a review and synthesis [J]. Sleep Med Rev.

[29] Teychenne M, Ball K, Salmon J (2008). Physical activity and likelihood of depression in adults: a review [J]. Prev Med.

[30] Lin HX, Liu Z, Chang C (2020). The effects of smoke-free workplace policies on individual smoking behaviors in China [J]. Nicotine tobacco Res.

[31] Radlof LS (1977). The CES-D scale a self-report depression scale for research in the general population [J]. Appl Psychol Meas.

[32] He J, Chen Z, Guo F, et al. Developing a Chinese Short Version of the Center for Epidemiologic Studies Depression Scale. Proc Int Convertion Psychol Sci F. 2015. [C].

[33] COMMITTEE I R. Guidelines for the Data Processing and Analysis of the International Physical Activity Questionnaire [EB/OL]. 2005. https://sites.google.com/site/theipaq. Accessed on 11 Jul 2019.

[34] Patterson E (2005). Guidelines for Data Processing and Analysis of the International Physical Activity Questionnaire (IPAQ)-Short and Long Forms [J].

[35] Mekary RA, Willett WC, Hu FB (2009). Isotemporal substitution paradigm for physical activity epidemiology and weight change [J]. Am J Epidemiol.

[36] Kraut R, Patterson M, Lundmark V (1998). Internet paradox. A social technology that reduces social involvement and psychological well-being? [J]. Am Psychol.

[37] Prince SA, Elliott CG, Scott K (2019). Device-measured physical activity, sedentary behaviour and cardiometabolic health and fitness across occupational groups: a systematic review and meta-analysis [J]. Int J Behav Nutr Phys Act.

[38] Smith L, Mccourt O, Sawyer A (2016). A review of occupational physical activity and sedentary behaviour correlates [J]. Occup Med.

[39] Mccrady SK, Levine JA (2009). Sedentariness at work: how much do we really sit? [J]. Obesity.

[40] Ekelund U, Steene-Johannessen J, Brown WJ (2016). Does physical activity attenuate, or even eliminate, the detrimental association of sitting time with mortality? A harmonised meta-analysis of data from more than 1 million men and women [J]. Lancet.

[41] Bartholomew LK, Markham CM, Ruiter RAC, et al. Planning health promotion programs: an intervention mapping approach [M]. Planning Health Promotion Programs: An Intervention Mapping Approach. 2016.

[42] Helgadóttir B, Hallgren M, Ekblom Ö (2016). Training fast or slow? Exercise for depression: a randomized controlled trial [J]. Prev Med.

[43] Cramer H, Lauche R, Langhorst J (2013). Yoga for depression: a systematic review and meta-analysis [J]. Depress Anxiety.

[44] Walmsley R, Chan S, Smith-Byrne K, et al. Reallocating time from machine-learned sleep, sedentary behaviour or light physical activity to moderate-to-vigorous physical activity is associated with lower cardiovascular disease risk [J]. Cold Spring Harbor Laboratory Press. 2020,

[45] Craft LL, Perna FM (2004). The benefits of exercise for the clinically depressed [J]. Prim Care Companion J Clin Psychiatry.

[46] Schuch FB, Vancampfort D, Richards J (2016). Exercise as a treatment for depression: a meta-analysis adjusting for publication bias [J]. J Psychiatr Res.

[47] Van Mill JG, Vogelzangs N, Van Someren EJ (2014). Sleep duration, but not insomnia, predicts the 2-year course of depressive and anxiety disorders [J]. J Clin Psychiatry.

[48] Van Noorden MS, Van Fenema EM, Van Der Wee NJ (2012). Predicting outcome of depression using the depressive symptom profile: the Leiden Routine Outcome Monitoring Study [J]. Depress Anxiety.

[49] Pigeon WR, Hegel M, Unützer J (2008). Is insomnia a perpetuating factor for late-life depression in the IMPACT cohort? [J]. Sleep.

[50] Stranges S, Dorn JM, Shipley MJ (2008). Correlates of short and long sleep duration: a cross-cultural comparison between the United Kingdom and the United States: the Whitehall II Study and the Western New York Health Study [J]. Am J Epidemiol.

